# SOX12 Promotes Stem Cell-Like Phenotypes and Osteosarcoma Tumor Growth by Upregulating JAGGED1

**DOI:** 10.1155/2021/9941733

**Published:** 2021-10-23

**Authors:** Weifei Zhang, Fei Yu, Jian Weng, Yien Zheng, Jianjing Lin, Tiantian Qi, Yihao Wei, Deli Wang, Hui Zeng

**Affiliations:** ^1^Department of Bone and Joint Surgery, Peking University Shenzhen Hospital, Shenzhen, China; ^2^National & Local Joint Engineering Research Center of Orthopedic Biomaterials, Peking University Shenzhen Hospital, Shenzhen 518036, China

## Abstract

SOX12 plays a role in promoting the growth of some tumors; however, its role in osteosarcoma remains unclear. From gene expression omnibus (GEO) and tumor alterations relevant for genomics-driven therapy (TARGET) databases, Kaplan–Meier analyses were conducted to establish relationships between SOX12 expression and osteosarcoma survival and recurrence in osteosarcoma patients. We also performed *in vitro* and *in vivo* assays to determine SOX12 function in osteosarcoma etiology. SOX12 expression was increased in osteosarcoma; high SOX12 expression levels were related to a poor prognosis and a high disease recurrence in patients. Moreover, SOX12 expression in osteosarcoma cell lines was increased, similar to osteosarcoma cancer stem cells. We also observed that SOX12 knockdown inhibited the spheroidization and expression of stemness markers in osteosarcoma cells and tumor formation in nude mice. In addition, SOX12 knockdown inhibited JAGGED1 and HES1 expression. Similarly, JAGGED1 knockdown also inhibited the formation of osteosarcoma cancer stem cells into pellets and reduced the expression of stemness markers and tumor formation capabilities in nude mice. Finally, during SOX12 knockdown, JAGGED1 overexpression rescued osteosarcoma cells from spheroidizing. SOX12 promotes stem cell-like phenotypes and osteosarcoma tumor growth by upregulating JAGGED1.

## 1. Introduction

Osteosarcoma is the most common malignant tumor in adolescents; the 5-year survival rate for patients with osteosarcoma, without metastasis, is approximately 70%, but that for those with osteosarcoma, with metastasis, is 20% [1]. Therefore, treatment measures for patients with metastatic osteosarcoma are urgently required.

Recent studies have reported that SOX12 plays important roles in various tumors, e.g., SOX12 promotes liver cancer metastasis [2]. In addition, SOX12 promotes the cell proliferative metastasis of colorectal cancer cells by regulating asparagine synthesis [3]. Similarly, SOX12 promotes metastasis in gastric cancer cells [4]. Equally, other studies have reported that SOX12 exerts inhibitory functions toward colon cancer metastasis [5]. However, the precise role of SOX12 in osteosarcoma remains unknown.

Cancer stem cells exist in small sections of tumors, have self-renewing proliferation and differentiation of cell populations, and are important causes of tumor metastasis, recurrence, and drug resistance [6]. Cancer stem cell regulation is performed via several key signaling pathways, e.g., Wnt/*β*-catenin, Notch, and Hedgehog [7]. JAGGED1 is the Notch ligand and plays key roles in cancer stem cell differentiation and metastasis; HES1 is a downstream molecule of JAGGED1 [8, 9]. For example, JAGGED1-Notch1-deployed tumor perivascular niches promote breast cancer stem cell phenotypes via Jag1-Notch1-Zeb1-VEGFA signal [10]. JAGGED1 also has important roles in lung cancer stem cells; it promotes stem cell phenotypes and the tumor growth of lung adenocarcinoma [11]. Importantly, JAGGED1 also has a key role in osteosarcoma cancer stem cells; it promotes stem cell-like phenotypes and osteosarcoma tumor growth [12].

In this study, we observed that SOX12 promoted stem cell-like phenotypes and osteosarcoma tumor growth by upregulating JAGGED1. Our data provides new insights for potential osteosarcoma treatments in clinical settings.

## 2. Materials and Methods

### 2.1. Plasmids and Reagents

SOX12-short hairpin (sh) RNA was purchased from HANBIO (Shanghai China). The target sequence was CATGGCGGATTACCCGGACTA [2]. Small interfering (si) RNA against JAGGED1 was obtained from RiboBio (Guangzhou, China). Pc-cDNA for SOX12 was purchased from IGE BIOTECHNOLOGY gene (Guangzhou, China). Pc-cDNA for JAGGED1 was purchased from BOMING gene (Guangzhou, China). MG63 and 143B osteosarcoma cell lines were obtained from the Cell Type Culture Collection of the Chinese Academy of Sciences (Shanghai, China). DMEM and fetal bovine serum (FBS, Gibco, Logan, USA) were used to culture cells. The JAGGED1 and *β*-actin antibody was purchased from ABclonal (A12733, China).

### 2.2. Clinical Specimens

SOX12 expression data were obtained from GSE28424 and GSE42352 databases from the gene expression omnibus (GEO) database (https://www.ncbi.nlm.nih.gov/geo/). Prognostic data for SOX12 were obtained from the GSE21257 database. Recurrence SOX12 data were obtained from the tumor alterations relevant for genomics-driven therapy (TARGET) database (https://ocg.cancer.gov/).

### 2.3. Cell Transfection

Lentivirus particles were directly added to cells in six-well plates. After 48 h, puromycin was added, after which culture medium and dead cells were aspirated. New complete medium was then added. Cells were grown until confluent. The siRNA and pc-cDNA and osteosarcoma cells were inoculated into a six-well plate. They were starved using Opti-MEM (Gibco) for 6 h. Lipofectamine 3000 (Invitrogen, USA) was then mixed with JAGGED1 siRNA and JAGGED1 pc-cDNA for 10 min, and cells were cultured with different mixtures for 6 h.

### 2.4. Real-Time Quantitative PCR

First, cellular RNA was extracted from cells using Trizol and 200 *μ*l chloroform added. The mixture was shaken and centrifuged at 12,000 rpm for 15 min. Next, an equal volume of isopropanol was added and finally dissolved in DEPC water. Then, real-time quantitative PCR (RT-qPCR) and cDNA synthesis were performed using a PrimeScript RT reagent kit (TaKaRa, Dalian, China). RT-qPCR was performed in triplicate using a SYBR Green PCR Master mix kit (TaKaRa) and an ABI Step-One system. Actin was used as an internal control. Data were analyzed with 2-*ΔΔ*ct method. Primers for RT-qPCR are listed (Table 1).

### 2.5. Western Blot Analysis

RIPA buffer was used to extract protein. Samples were centrifuged to generate supernatants, after which loading buffer was added to aliquots, and samples boiled for 10 min. Proteins were electrophoresed for 2 h and transferred to polyvinylidene fluoride membranes. A primary antibody was incubated with the membrane overnight, and the next day, a secondary antibody was added. Membranes were then photographed under a fluorescence microscope.

### 2.6. Tumor Sphere Assay

Cells were seeded at 1 × 10^3^ cells/well in six-well ultralow attachment plates (Corning Inc., Corning, NY, USA) in DMEM/F12 (Invitrogen, Carlsbad, CA) supplemented with N2 medium (Invitrogen), human EGF (10 ng/ml, Peprotech, USA), and human bFGF (10 ng/ml, Peprotech). After culturing for 14 days, the total number of spheres was counted.

### 2.7. Animal Studies

We used 4-week-old female BALA/c nude mice (6 per group) that were purchased from Guangdong Provincial Animal Medical Center. We injected 1 × 10^5^ cells under the skin, and subsequent tumor volumes were calculated using the formula *V* = 1/2 (width^2^ × length). Mice were sacrificed on day 40 after injection. Tumor size and number were analyzed.

### 2.8. Statistical Analysis

Outcomes were compared using Student's *t*-test. Prognostic comparisons were performed using the Kaplan–Meier method. Survival curves were compared using the log-rank test. All statistical data were analyzed using SPSS 19.0 (IBM, Chicago, IL, USA) and GraphPad Prism 7 (La Jolla, CA, USA).

## 3. Results

### 3.1. SOX12 Is Highly Expressed in Osteosarcoma; High SOX12 Expression Levels Are Associated with a Poor Prognosis and a High Disease Recurrence Rate in Patients with Osteosarcoma

**Figure 1:**
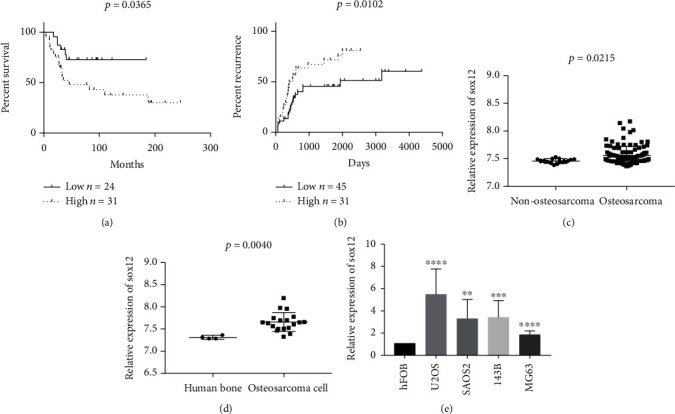
Clinical information for SOX12. (a) Kaplan–Meier survival curves of low and high SOX12 levels in the GSE21257 database. (b) Kaplan–Meier analyses reveal correlations between SOX12 expression and tumor recurrence in the TARGET database. (c) Relative expression of SOX12 in human bone and osteosarcoma cells in the GSE42352 database. (d) Relative expression of SOX12 in nonosteosarcoma and osteosarcoma in the GSE28424 database. (e) Relative mRNA expression of SOX12 in osteosarcoma cell lines compared to hFOB. ^∗∗^*p* < 0.01, ^∗∗∗^*p* < 0.001, ^∗∗∗∗^*p* < 0.0001.

To explore the relationship between SOX12 expression and osteosarcoma prognosis or recurrence, we analyzed the GSE21257 database (Table 2) and observed that SOX12 elevated expression indicated a poor prognosis for patients with osteosarcoma (Figure 1(a)). From the TARGET database (Table 3), patients with osteosarcoma expressing high SOX12 levels had higher disease recurrence rates (Figure 1(b)). Furthermore, SOX12 expression in osteosarcoma cell lines was higher than normal bone tissue in the GSE42352 database (Figure 1(c)). Similarly, from the GSE28424 database, SOX12 expression in osteosarcoma patients was higher than in nonosteosarcoma patients (Figure 1(d)). SOX12 expression was highly regulated in the osteosarcoma cell lines, U2OS, SAOS2, 143B, and MG63, when compared with human osteoblasts, hFOB (Figure 1(e)).

### 3.2. SOX12 Is Highly Expressed in Osteosarcoma Stem Cells

**Figure 2:**
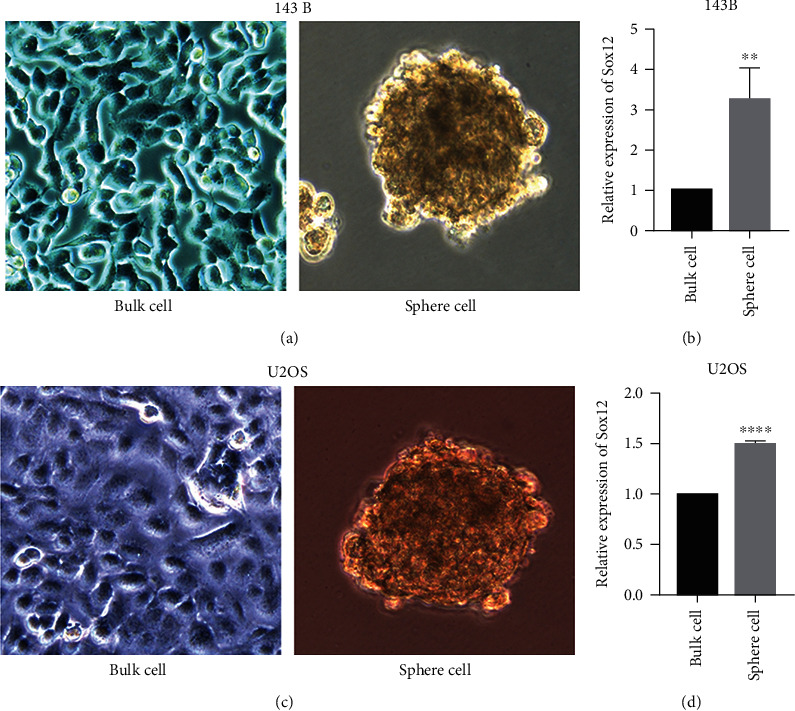
Osteosarcoma CSCs express high SOX12 levels. (a) Spheres formed under these culture conditions (osteosarcoma CSCs), as well as monolayer cells cultured under the regular culture conditions (143B bulk cells). (b) The relative mRNA expression of SOX12 in osteosarcoma CSCs and 143B bulk cells. (c) Spheres formed under these culture conditions (osteosarcoma CSCs), as well as monolayer cells cultured under the regular culture conditions (U2OS bulk cells). (d) The relative mRNA expression of SOX12 in osteosarcoma CSCs and U2OS bulk cells. ^∗∗^*p* < 0.01, ^∗∗∗∗^*p* < 0.0001.

We assessed SOX12 expression in osteosarcoma stem cells. In the absence of serum, cells were suspended into spheres [13, 14]. These cells can be enriched in sphere cultures under the conditions of osteosarcoma stem cell culture; we grew two osteosarcoma cell lines 143B and U2OS under sphere culture conditions to enrich CSCs, and then, we detect osteosarcoma the expression of SOX12 in stem cells and ordinary cells. SOX12 expression in osteosarcoma stem cells was higher (Figures 2(a) and 2(b)). The same was observed for U2OS cells (Figures 2(c) and 2(d)), suggesting that highly expressed SOX12 was critical for the maintenance of CSCs.

### 3.3. SOX12 Expression in Cells Transfected with Lentivirus Is Decreased

**Figure 3:**
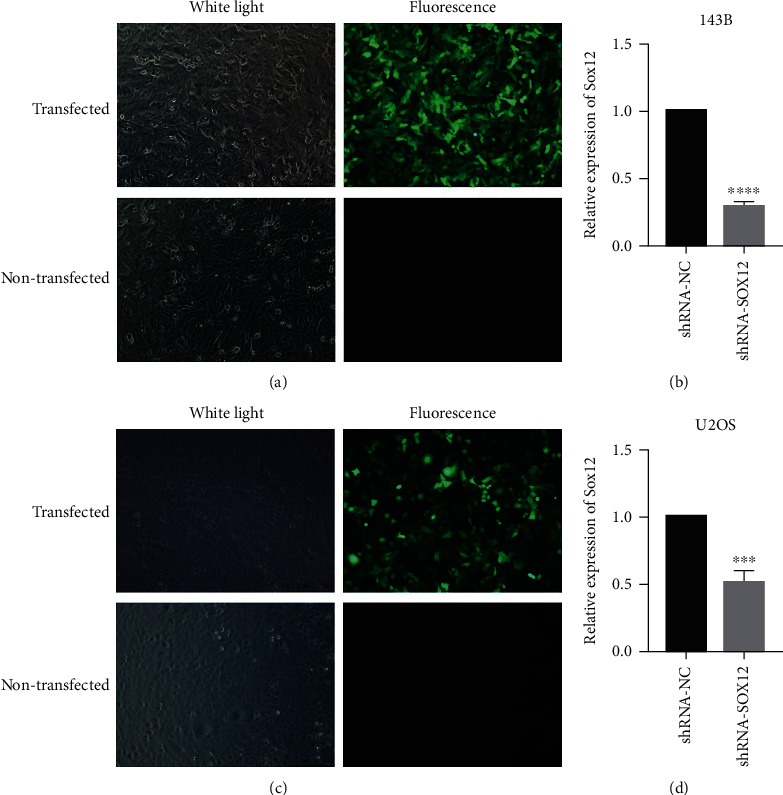
SOX12 expression decreases in cells transfected with lentivirus. (a) Transfected and untransfected 143B cells under white light and fluorescence. (b) SOX12 expression in 143B cells transfected with shRNA-NC and shRNA-SOX12. (c) Transfected and untransfected U2OS cells under white light and fluorescence. (d) SOX12 expression in U2OS cells transfected with shRNA-NC and shRNA-SOX12. ^∗∗∗^*p* < 0.001, ^∗∗∗∗^*p* < 0.0001.

We transfected lentivirus expressing green fluorescent protein into cells and fluorescently imaged cells after screening with puromycin. Transfected cells exhibited fluorescence, while untransfected cells exhibited none (Figure 3(a)). SOX12 expression in osteosarcoma cells transfected with shRNA-SOX12 was downregulated when compared with cells transfected with shRNA-NC (Figure 3(b)). This also occurred in U2OS cells (Figures 3(c) and 3(d)).

### 3.4. SOX12 Downregulation Inhibits the Stem Cell-Like Properties of Osteosarcoma Cells

**Figure 4:**
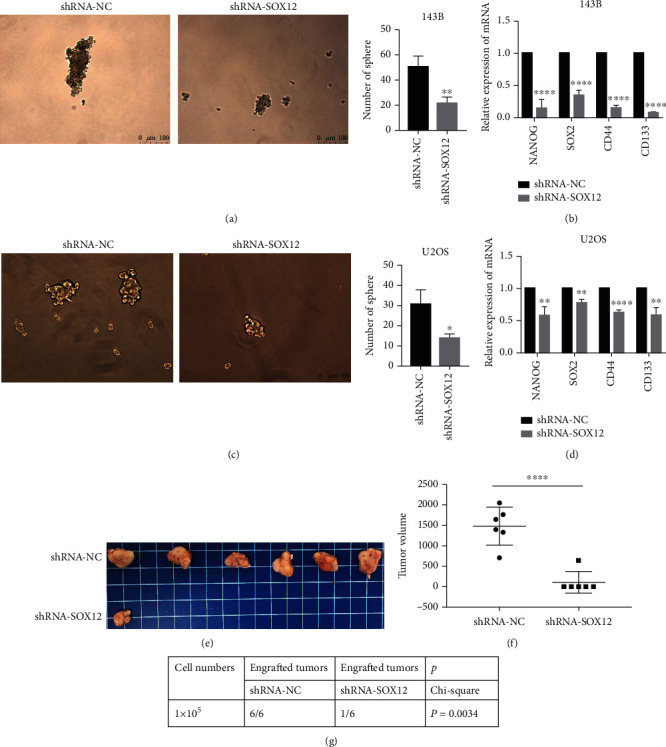
SOX12 downregulation inhibits the stem cell-like properties of osteosarcoma cells. (a) SOX12 knockdown inhibits the spheroidizing ability of 143B cells. (b) SOX12 knockdown reduces stemness marker expression in 143B cells. (c) SOX12 knockdown inhibits spheroidizing abilities in U2OS cells. (d) SOX12 knockdown reduces stemness marker expression in U2OS cells. (e–g) SOX12 knockdown inhibits tumor size and numbers in nude mice. ^∗^*p* < 0.01, ^∗∗^*p* < 0.001, ^∗∗∗∗^*p* < 0.0001.

After osteosarcoma cells were transfected with shRNA-SOX12 lentivirus, their ability to form spheroids was decreased, which was manifested in that the osteosarcoma cells that knock down SOX12 form less spheroids; the number of balls in the spheroidization experiment represents the stemness of tumor cells; this means that knocking down SOX12 can inhibit the stemness of osteosarcoma stem cells (Figures 4(a) and 4(c)). In addition, the molecular stemness markers, NANOG, SOX2, CD44, and CD133, were also reduced (Figures 4(b) and 4(d)). From our *in vivo* stemness assay, where we injected 1 × 10^5^ 143B osteosarcoma cells mixed with 100 *μ*l DMEM into nude mice, we observed that after 40 days, only one of the knockdown SOX12 group had successfully grown a tumor, while all in the NC group had a tumor (Figures 4(e)–4(g)).

### 3.5. SOX12 Overexpression Promotes the Stem Cell-Like Properties of Osteosarcoma Cells

**Figure 5:**
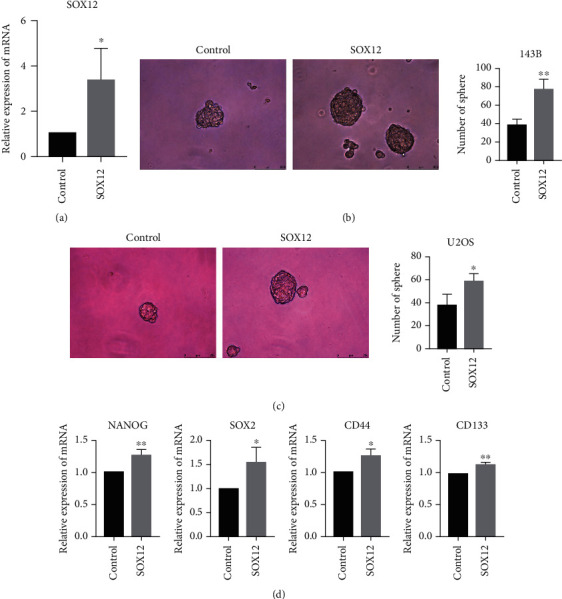
SOX12 overexpression promotes the stem cell-like properties of osteosarcoma cells. (a) Transfection of SOX12 plasmid increased SOX12 mRNA in 143B cells. (b) Overexpression of SOX12 enhances the spheroidizing ability of 143B cells. (c) Overexpression of SOX12 enhances the spheroidizing ability of U2OS cells. (d) Overexpression of SOX12 upregulated the stemness markers of 143B cells.

By transfecting a plasmid overexpressing SOX12, the efficiency of transfection in 143B cells was verified by PCR (Figure 5(a)). After that, we conducted tumor stemness-related assays, and we found that the osteosarcoma cells overexpressing SOX12 are more capable of forming spheres than the control (Figures 5(b) and 5(c)), and the stemness markers of the 143B cells overexpressing SOX12 are higher than that of the control group (Figure 5(d)).

### 3.6. SOX12 Knockdown Reduces JAGGED1 and HES1 Expression

**Figure 6:**
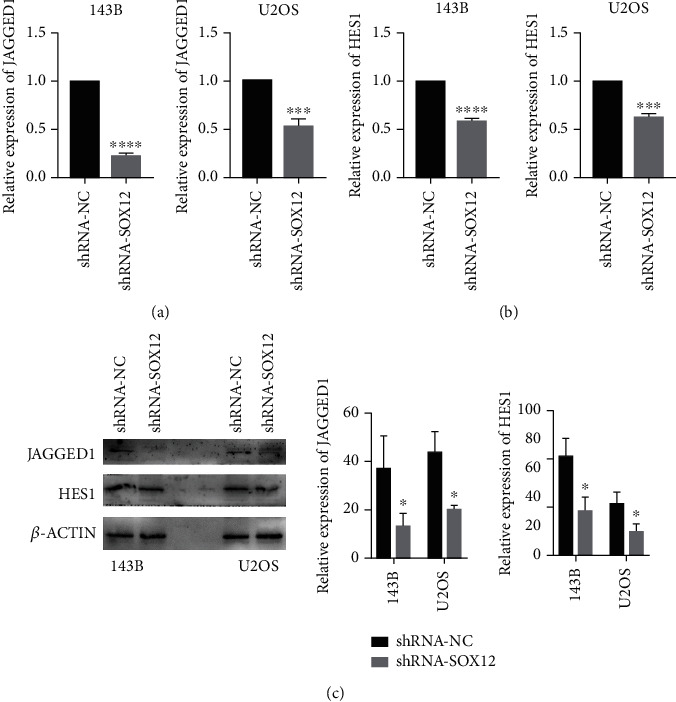
SOX12 knockdown reduces JAGGED1 and HES1 expression. (a) SOX12 knockdown in osteosarcoma cells inhibits JAGGED1 mRNA expression. (b) SOX12 knockdown in osteosarcoma cells inhibits HES1 mRNA expression. (c) SOX12 knockdown in osteosarcoma cells inhibits JAGGED1 and HES1 protein expression. ^∗^*p* < 0.01, ^∗∗∗^*p* < 0.001, ^∗∗∗∗^*p* < 0.0001.

From the literature, we observed (Supplementary Table S3) that stem-related molecule after overexpression SOX12 is JAGGED1 [2]; thus, we investigated JAGGED1 mRNA and protein expression by RT-qPCR and Western blotting. We observed that JAGGED1 mRNA and protein expression were suppressed after SOX12 knockdown. Similarly, the mRNA and protein of HES1 are also reduced (Figures 6(a)–6(c)).

### 3.7. JAGGED1 Inhibition Reduces Stemness Abilities in Osteosarcoma Cells

**Figure 7:**
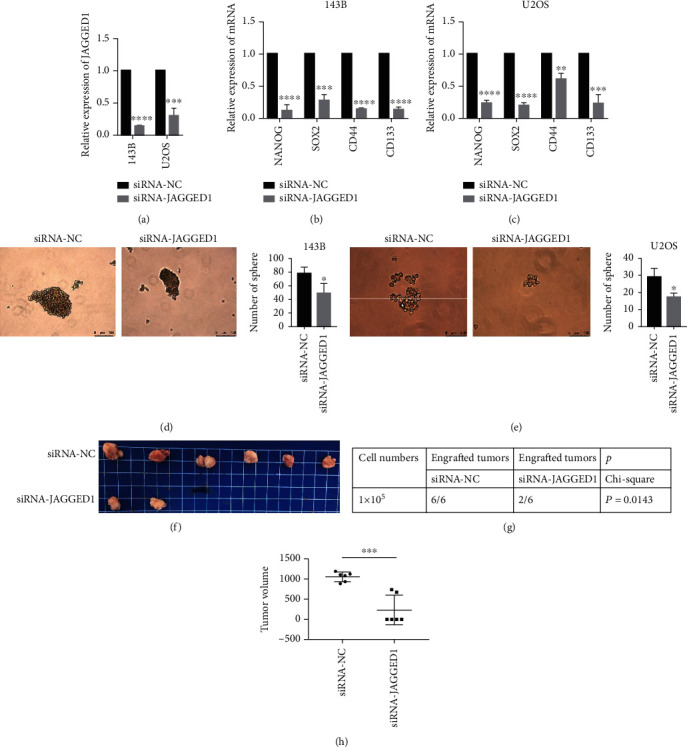
JAGGED1 downregulation inhibits the stem cell-like properties of osteosarcoma cells. (a) JAGGED1 knockdown reduced its mRNA expression. (b, c) JAGGED1 knockdown reduces stemness marker levels in osteosarcoma cells. (d, e) JAGGED1 knockdown inhibits the spheroidizing ability of osteosarcoma cells. (f–h) JAGGED1 knockdown inhibits tumor size and numbers in nude mice. ^∗^*p* < 0.05, ^∗∗^*p* < 0.01, ^∗∗∗^*p* < 0.001, ^∗∗∗∗^*p* < 0.0001.

JAGGED1 is a key factor in promoting cancer stem cells [15]. We observed that siRNA-mediated JAGGED1 inhibition reduced JAGGED1 expression (Figure 7(a)). Our RT-qPCR data revealed that mRNA levels of stemness-related markers, such as NANOG, SOX2, CD44, and CD133, were significantly downregulated in JAGGED1-silenced osteosarcoma cells (Figures 7(b) and 7(c)). Moreover, the spheroidization ability of osteosarcoma stem cells transfected with siRNA-JAGGED1 was less than osteosarcoma stem cells transfected with siRNA-NC (Figures 7(d) and 7(e)). In addition, our *in vivo* stemness assay after day 40 indicated that one of the JAGGED1 knockdown groups successfully grew two tumors, while all in the NC group had one tumor (Figures 7(f)–7(h)).

### 3.8. JAGGED1 Overexpression Partially Rescues the Spheroidizing Ability of Osteosarcoma Cells Transfected with shRNA-SOX12

**Figure 8:**
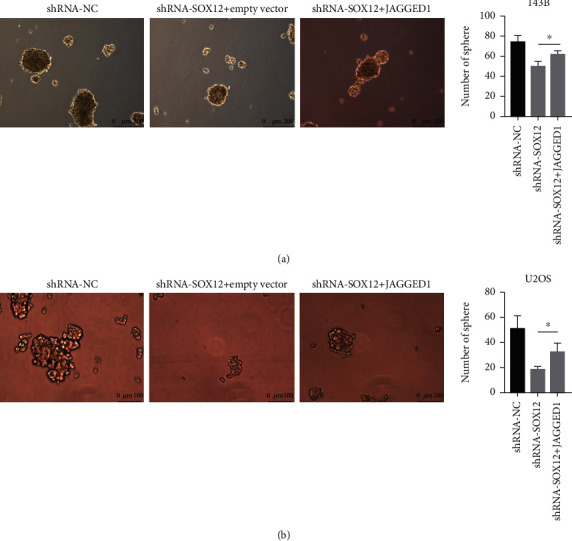
JAGGED1 overexpression partially rescues the spheroidizing ability of osteosarcoma cells. (a) JAGGED1 overexpression partially rescues the spheroidizing ability of 143B osteosarcoma cells. (b) JAGGED1 overexpression partially rescues the spheroidizing ability of U2OS osteosarcoma cells. ^∗^*p* < 0.05.

We investigated whether SOX12 functioned via JAGGED1 interaction. By overexpressing JAGGED1 in cells transfected with shRNA-SOX12, when compared with empty plasmid transfected cells, JAGGED1 overexpression partially rescued the spheroidizing ability of osteosarcoma cells transfected with shRNA-SOX12 (Figures 8(a) and 8(b)).

## 4. Discussion

SOX12 is a member of the SOX gene family and plays important roles in various biological processes, including tumor cell differentiation and immunity [16–19]. It also inhibits the progression of liver, stomach, and colorectal cancer [2–4]. However, its role in osteosarcoma is currently unknown. In our study, we observed that SOX12 promoted stem cell-like phenotypes and osteosarcoma tumor growth by upregulating JAGGED1.

The American Association for Cancer Research Stem Cell Workshop defines that cancer stem cells are a small part of cells with stem cell properties in tomor tissues; it has unlimited self-renewal ability and can produce the same progeny cells as the previous generation, and it has a variety of differentiation potentials and high growth reproductive ability, and it can form tumor tissue composed of heterogeneous tumor cells [20]. Many researchers believe that cancer stem cells are derived from mutations in normal tissue stem cells, with some believe they are derived from somatic mutations. Cancer stem cells typically have the following characteristics: self-renewal abilities, high tumorigenicity, differentiation potential, and drug resistance [21, 22]. Cancer stem cell markers also include NANOG, ALDH1, SOX2, OCT4, LGR5, CD44, and CD133; therefore, stemness may be judged by the expression of these markers [23].

The sphere formation assay is a common sorting method for osteosarcoma cancer stem cells [14]. Osteosarcoma stem cells are more capable of tumor formation in nude mice than ordinary osteosarcoma cells [24]. The most important thing is that studies have pointed out that the existence of osteosarcoma stem cells is an important cause of tumor metastasis, drug resistance, and recurrence. Therefore, the development of therapies for tumor stem cells has become an important tumor treatment goal [25].

The NOTCH signaling pathway promotes the occurrence and development of cancer stem cells [26]. JAGGED1 is a notch ligand and plays a key role in cancer stem cells; HES1 is a downstream molecule of JAGGED1 [11, 27]. Similarly, JAGGED1 also plays an important role in osteosarcoma stem cells, e.g., the microRNA; miR-26a inhibits stem cell-like phenotypes and osteosarcoma tumor growth by targeting JAGGED1 [12]. Therefore, JAGGED1 may be an important target for cancer stem cells [28].

SOX12 has important roles in many tumors; a previous study indicated that SOX12 is a novel potential target for acute myeloid leukemia [29]. In addition, it promotes the growth and metastatic potential of triple-negative breast cancer cells [30]. Studies have also indicated that SOX12 is an important marker of liver cancer stem cells [31, 32], whereas other studies have suggested that it promotes stemness in glioma cells [33]. Therefore, it is accepted that SOX12 plays key roles in many tumor types and some tumor stem cells.

Our most important finding for our study was that SOX12 was highly expressed in osteosarcoma, and this high expression was related to a poor prognosis and high disease recurrence in osteosarcoma patients. When compared with ordinary osteosarcoma cells, SOX12 expression in osteosarcoma cancer stem cells was increased. After SOX12 knockdown, the spheroidization ability of osteosarcoma cells was decreased. Stemness marker expression and tumorigenic abilities in nude mice also decreased. Similarly, after SOX12 knockdown, JAGGED1 and HES1 expression also decreased. We then knocked down JAGGED1 expression with siRNA and observed that spheroidizing capability, the expression of dry molecular markers, and tumorigenic abilities in nude mice decreased. Finally, by knocking down SOX12 and overexpressing JAGGED1, the spheroidizing ability of cells was rescued.

Finally, we have shown that SOX12 promotes stem cell-like phenotypes and osteosarcoma tumor growth by upregulating JAGGED1. Our data indicates that these molecules could provide novel molecular therapeutics for the treatment of osteosarcoma.

## 5. Conclusions

SOX12 promotes stem cell-like phenotypes and osteosarcoma tumor growth by upregulating JAGGED1. Our data indicates that these molecules could provide novel molecular therapeutics for the treatment of osteosarcoma.

## Figures and Tables

**Table 1:** The primers for RT-qPCR.

Gene	Sequence (5-3)
SOX12	F: GACATGCACAACGCCGAGATCT
	R: GTAATCCGCCATGTGCTTGAGC
JAGGED1	F: TGCTACAACCGTGCCAGTGACT
	R: TCAGGTGTGTCGTTGGAAGCCA
ACTIN	F: CACCATTGGCAATGAGCGGTTC
	R: AGGTCTTTGCGGATGTCCACGT
NANOG	F: ATGGAGGAGGGAAGAGGAGA
	R: GATTTGTGGGCCTGAAGAAA
SOX2	F: GCTTAGCCTCGTCGATGAAC
	R: AACCCCAAGATGCACAACTC
CD44	F: CGTGGAATACACCTGCAAAG
	R: CGGACACCATGGACAAGTTT
CD133	F: TTTTGGATTCATATGCCTTCTGT
	R: ACCCATTGGCATTCTCTTTG
HES1	F: GGAAATGACAGTGAAGCACCTCC
	R: GAAGCGGGTCACCTCGTTCATG

**Table 2:** Association between SOX12 expression and clinicopathological parameters in GSE21257.

Parameters	SOX12 high (*n* = 27)	SOX12 low (*n* = 26)	X2	*p*
Gender				
Male	19	15	0.926	0.336
Female	8	11		
Age (months)				
≥180	19	15	0.926	0.336
<180	8	11		
Subtype				
Osteoblastic	20	12	4.316	0.038∗
Nonosteoblastic	7	14		
Location				
Femur	14	13	0.077	0.781
Nonfemur	12	13		
Unknown	1			
Metastases				
Yes	19	15	0.926	0.336
No	8	11		

**Table 3:** Association between SOX12 expression and clinicopathological parameters in the TARGET database.

Parameters	SOX12 high (*n* = 31)	SOX12 low (*n* = 45)	X2	*p*
Gender				
Male	22	21	2.101	0.036∗
Female	9	24		
Metastases				
Yes	9	10	0.674	0.500
No	22	35		
Location				
Femur	12	22	0.877	0.380
Nonfemur	19	23		

## Data Availability

The data used to support the findings of this study are included within the article.

## Supplementary Materials

Supplementary Materials Supplementary Table S3: list of genes differentially expressed in Huh7-Sox12 versus Huh7-control cells using a human EMT PCR array.
