# Supplementary material for: SOX12 Promotes Stem Cell-Like Phenotypes and Osteosarcoma Tumor Growth by Upregulating JAGGED1
Source: Stem Cells Int. 2021 Oct 23;2021:9941733. doi: 10.1155/2021/9941733 (PMC8557074; doi:10.1155/2021/9941733)
Supplement: Supplementary Materials — Supplementary Table S3: list of genes differentially expressed in Huh7-Sox12 versus Huh7-control cells using a human EMT PCR array. [file 9941733.f1.doc]

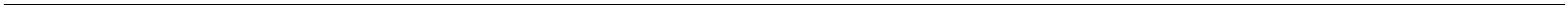

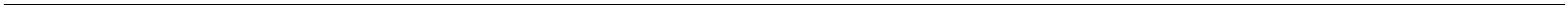
Supplementary Table S3. List of genes differentially expressed in Huh7-Sox12 versus

Huh7-control cells using a human EMT PCR array

Symbol fold change Description

FGFBP1 5.28 Fibroblast growth factor binding protein 1

TWIST1 4.62 Twist homolog 1 (Drosophila)

JAG1 4.42 Jagged 1

MST1R(RON) 4.11 Macrophage stimulating 1 receptor

ZEB2 3.96 Zinc finger E-box binding homeobox 2

ITGAV 3.75 Integrin, alpha V (vitronectin receptor)

VIM 3.58 Vimentin

MMP9 3.39 Matrix metallopeptidase 9

ERBB3 3.21 V-erb-b2 erythroblastic leukemia viral oncogene homolog 3

FN1 2.97 Fibronectin 1

ITGA5 2.69 Integrin, alpha 5 (fibronectin receptor, alpha polypeptide)

ZEB1 2.52 Zinc finger E-box binding homeobox 1

CDH2 2.41 Cadherin 2, type 1, N-cadherin (neuronal)

FOXC2 2.35 Forkhead box C2 (MFH-1, mesenchyme forkhead 1)

COL5A2 2.29 Collagen, type V, alpha 2

NODAL 2.18 Nodal homolog (mouse)

CTNNB1 2.08 Catenin (cadherin-associated protein), beta 1, 88kDa

MSN 1.96 Moesin

MMP2 1.88 Matrix metallopeptidase 2

SNAI2(Slug) 1.85 Snail homolog 2 (Drosophila)

NOTCH1 1.77 Notch 1

TGFB1 1.74 Transforming growth factor, beta 1

TGFB2 1.71 Transforming growth factor, beta 2

COL3A1 1.69 Collagen, type III, alpha 1

TCF4 1.67 Transcription factor 4

GSK3B 1.55 Glycogen synthase kinase 3 beta

SNAI1 1.46 Snail homolog 1 (Drosophila)

BMP2 1.45 Bone morphogenetic protein 2

RAC1 1.44 Ras-related C3 botulinum toxin substrate 1 (rho family)

CAV2 1.44 Caveolin 2

PTP4A1(PRL1) 1.41 Protein tyrosine phosphatase type IVA, member 1

ITGB1 1.38 Integrin, beta 1 (fibronectin receptor)

PDGFRB 1.35 Platelet-derived growth factor receptor, beta polypeptide

MAP1B 1.33 Microtubule-associated protein 1B

PTK2(FAK) 1.33 PTK2 protein tyrosine kinase 2

SMAD2 1.33 SMAD family member 2

BMP7 1.32 Bone morphogenetic protein 7

COL1A2 1.28 Collagen, type I, alpha 2

TCF3 1.25 Transcription factor 3

KRT14 1.23 Keratin 14

B2M 1.22 Beta-2-microglobulin

AHNAK 1.19 AHNAK nucleoprotein

OCLN 1.17 Occludin

SNAI3 1.17 Snail homolog 3 (Drosophila)

MMP3 1.16 Matrix metallopeptidase 3 (stromelysin 1, progelatinase)

TGFB3 1.15 Transforming growth factor, beta 3

FZD7 1.14 Frizzled family receptor 7

GEMIN2 1.12 Survival of motor neuron protein interacting protein 1

WNT11 1.1 Wingless-type MMTV integration site family, member 11

WNT5A 1.08 Wingless-type MMTV integration site family, member 5A

ILK 1.07 Integrin-linked kinase

SOX10 1.05 SRY (sex determining region Y)-box 10

STAT3 1.05 Signal transducer and activator of transcription 3

AKT1 1.04 V-akt murine thymoma viral oncogene homolog 1

ESR1(ERa) 1.01 Estrogen receptor 1

KRT19 -1.03 Keratin 19

EGFR -1.05 Epidermal growth factor receptor

HPRT1 -1.07 Hypoxanthine phosphoribosyltransferase 1

GSC -1.09 Goosecoid homeobox

CALD1 -1.12 Caldesmon 1

KRT7 -1.16 Keratin 7

STEAP1 -1.21 Six transmembrane epithelial antigen of the prostate 1

F11R -1.22 F11 receptor

TMEFF1 -1.26 Transmembrane protein with EGF-like and two follistatin-like domains 1

VCAN -1.28 Versican

WNT5B -1.32 Wingless-type MMTV integration site family, member 5B

IL1RN -1.34 Interleukin 1 receptor antagonist TMEM132A -1.37 Transmembrane protein 132A

DESI1 -1.38 PPPDE peptidase domain containing 2

BMP1 -1.44 Bone morphogenetic protein 1

GNG11 -1.56 Guanine nucleotide binding protein (G protein), gamma 11

PLEK2 -1.54 Pleckstrin 2

VPS13A -1.59 Vacuolar protein sorting 13 homolog A (S. cerevisiae)

TSPAN13 -1.71 Tetraspanin 13 DSP -1.85 Desmoplakin

SPARC -1.86 Secreted protein, acidic, cysteine-rich (osteonectin)

RGS2 -1.91 Regulator of G-protein signaling 2, 24kDa

NUDT13 -1.91 Nudix (nucleoside diphosphate linked moiety X)-type motif 13

SERPINE1 -2.11 Serpin peptidase inhibitor, clade E member 1

TIMP1 -2.18 TIMP metallopeptidase inhibitor 1 TFPI2 -2.26 Tissue factor pathway inhibitor 2

IGFBP4 -2.39 Insulin-like growth factor binding protein 4

DSC2 -2.87 Desmocollin 2

CAMK2N1 -3.12 Calcium/calmodulin-dependent protein kinase II inhibitor 1

CDH1 -3.91 Cadherin 1, type 1, E-cadherin (epithelial)


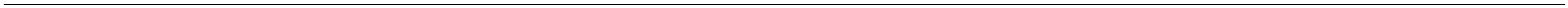
SPP1 -3.98 Secreted phosphoprotein 1
